# Sequencing flow-sorted short arm of *Haynaldia villosa* chromosome 4V provides insights into its molecular structure and virtual gene order

**DOI:** 10.1186/s12864-017-4211-7

**Published:** 2017-10-16

**Authors:** Jin Xiao, Keli Dai, Lian Fu, Jan Vrána, Marie Kubaláková, Wentao Wan, Haojie Sun, Jing Zhao, Chunyan Yu, Yufeng Wu, Michael Abrouk, Haiyan Wang, Jaroslav Doležel, Xiue Wang

**Affiliations:** 1 State Key Laboratory of Crop Genetics and Germplasm Enhancement, Cytogenetics Institute, Nanjing Agricultural University/JCIC-MCP, Nanjing, 210095 China; 2 Institute of Experimental Botany, Centre of the Haná Region for Biotechnological and Agricultural Research, Šlechtitelů 31, CZ-783671 Olomouc, Czech Republic

**Keywords:** *Haynaldia villosa*, Flow sorting, Chromosome arm 4VS, Scaffold, Genome zipper

## Abstract

**Background:**

*Haynaldia villosa* (*H. villosa*) has been recognized as a species potentially useful for wheat improvement. The availability of its genomic sequences will boost its research and application.

**Results:**

In this work, the short arm of *H. villosa* chromosome 4V (4VS) was sorted by flow cytometry and sequenced using Illumina platform. About 170.6 Mb assembled sequences were obtained. Further analysis showed that repetitive elements accounted for about 64.6% of 4VS, while the coding fraction, which is corresponding to 1977 annotated genes, represented 1.5% of the arm. The syntenic regions of the 4VS were searched and identified on wheat group 4 chromosomes 4AL, 4BS, 4DS, *Brachypodium* chromosomes 1 and 4, rice chromosomes 3 and 11, and sorghum chromosomes 1, 5 and 8. Based on genome-zipper analysis, a virtual gene order comprising 735 gene loci on 4VS genome was built by referring to the *Brachypodium* genome, which was relatively consistent with the scaffold order determined for *Ae. tauschii* chromosome 4D. The homologous alleles of several cloned genes on wheat group 4 chromosomes including *Rht-1* gene were identified.

**Conclusions:**

The sequences provided valuable information for mapping and positional-cloning genes located on 4VS, such as the wheat yellow mosaic virus resistance gene *Wss1*. The work on 4VS provided detailed insights into the genome of *H. villosa*, and may also serve as a model for sequencing the remaining parts of *H. villosa* genome.

**Electronic supplementary material:**

The online version of this article (10.1186/s12864-017-4211-7) contains supplementary material, which is available to authorized users.

## Background

The availability of genome sequences has facilitated breeding improved varieties in rice, sorghum and maize [1–3]. However, plant species with large and complex genomes, such as wheat and its relatives, remain a challenge for sequencing. To overcome the difficulties, two strategies have been applied in wheat genome. The first one relies on diploid and in some cases tetraploid progenitors as surrogates. Using this method, two diploid progenitors for wheat A and D sub-genomes *T. urartu* and *Ae. tauschii*, respectively, were sequenced [4–6]. While this method makes sequencing simplified to some extent, sequencing the complex diploid still encounters a tough challenge, as it either provides a large proportion of fragmented and unarranged genome sequences or is laborious and daunting. The second approach was proposed by Doležel et al. [7] based on flow-sorting individual chromosomes. Recently, International Wheat Genome Sequencing Consortium (IWGSC) has applied this chromosome-based strategy for sequencing the large size, highly repetitive and allohexaploidy wheat genome [8, 9].

Flow cytometric chromosome sorting may dramatically simplify genome analysis by reducing genome to controllable size. Unfortunately, sorting particular chromosomes may not be possible if they cannot be discriminated based on their fluorescence intensity or light scatter [10]. In wheat, only chromosome 3B can be easily sorted [11]. A solution has been used to sort chromosome arms from telosomic lines [12]. Efforts has been made by the IWGSC [8] to use a set of ditelosomic wheat lines for flow-sorting, sequencing, and de novo assembling each chromosome arm (except for 3B) of wheat genome. Recently, new strategies based on fluorescent labeled large microsatellite clusters enable us to differentiate and isolate chromosomes having similar DNA content [13, 14].

By wide hybridization and chromosome manipulation, alien chromosome addition lines involving different wild species have been produced. Alien chromosomes in wheat background can be purified by flow sorting if they are different from the host wheat chromosomes. This provides an elegant solution if a chromosome cannot be flow-sorted from its native species. Kubaláková et al. used a set of wheat-rye addition lines to isolate all seven rye chromosomes [15]. Similarly, a wheat alien chromosome addition line “T240” was used to isolate chromosome arm 6VS of *Haynaldia villosa* (*H. villosa*) [16].

Next-generation high-throughput DNA sequencing techniques (NGS) with remarkably improved sequencing capability provides opportunities to obtain a large amount of sequences in a very short time and at an acceptable low cost, and facilitated the availability of a draft genome reference for many plant species [17]. Although this method often generates a draft version in an organism sequencing project [4, 5, 18], these sequences obtained are informative for gene discovery, chromosome structure study, marker development and comparative studies. Moreover, the combination of NGS technology with single chromosome sorting approach have been demonstrated to dramatically improve the sequence quality for barley [19], rye [20], wheat chromosome 3B [9] and other wheat chromosomes [8, 21–24].

*H. villosa* (L.) Schur (syn. *Dasypyrum villosum* L. Candargy, 2n = 14, genome VV) is a wheat wild relative carrying many favorable genes for wheat improvement [25]. In previous study, a wheat yellow mosaic (WYM) resistance gene *Wss1* [26] and an eye-spot resistance gene [27] have been located on chromosome arm 4VS. By the development of various translocations involving 4VS using the *ph1b* induction system, *Wss1* was mapped to the distal region of 4VS [28]. The lack of *H. villosa* genome sequence hampers the cloning of favorite genes from *H. villosa*, including *Wss1*. In this study, a wheat alien chromosome addition line which contains a pair of short arms of chromosome 4V (4VS) of *H. villosa* was used to isolate, sequence and de novo assemble sequence of 4VS. The draft sequence obtained will be used to characterize the genomic composition of 4VS including repetitive sequences and gene content, identify microRNA (miRNA) precursors and perform genome-zipper analysis to find syntenic regions among genomes of Triticeae homoeologous group 4 and other sequenced grasses. The sequences can also be used to develop cytogenetic and PCR-based 4VS specific markers [29], which have the potential use to trace and define alien chromosome in 4VS small fragment translocation lines. The 4VS survey sequence will provide an outline of genome features for *H. villosa* and facilitate candidate genes discovery on 4VS. The work will be extended to the remaining chromosomes of *H. villosa*.

## Methods

### Plant materials

The *H. villosa* (Accession No. 91C43, 2n = 14, VV) was introduced from Cambridge Botanical Garden, UK. *T. aestivum-H. villosa* ditelosomic addition line Dt4VS (Accession No. NAU1201) and disomic substitution line DS3V (Accession No. NAU352), were developed by the Cytogenetics Institute, Nanjing Agricultural University. Dt4VS represents a wheat genetic stock, in which except the 42 chromosomes of wheat, a pair of the short arm of *H. villosa* chromosomes 4V are added into wheat [the somatic cell chromosome constitution is 2n = [42(AABBDD) + 2 t(4VS)]. DS3V represents a wheat genetic stock, in which contain 40 of the 42 wheat chromosomes, and the pair of wheat chromosome 3D are substituted by *H. villosa* chromosome 3 V [the somatic cell chromosome constitution is 2n = [40(AABBDD-3D3D) + 3V3V].

### Chromosome sorting and DNA sequencing

Aqueous suspensions of chromosome 4VS of *H. villosa* were prepared from synchronized meristem root tip cells following Vránaet al. [11] and Kubaláková et al. [12]. The chromosomes in suspension were stained with 2 μg/ml 4′, 6-diamidino-2-phenylindole (DAPI) and the 4VS telosomes were sorted using a FACSVantage SE flow cytometer and sorter (Becton Dickinson, San Jose, USA). Purity in the sorted fractions was determined after fluorescence in situ hybridization (FISH) with two probes (microsatellite GAA and p*Sc119.*2) on sorted chromosomes spread on the microscope slides. DNA of the sorted chromosome arms was purified and amplified by multiple displacement amplification (MDA) using the illustraTMGenomiPhi V2 DNA Amplification Kit (GE Healthcare Bio-Sciences Corp., Piscataway, NJ, USA) as described by Šimková et al. [30]. Three independent amplification products were combined to reduce amplification bias. The amplified DNA was purified by ethanol precipitation before sequencing.

About 10 μg of MDA-amplified DNA was used to create the two shotgun DNA-seq libraries of 500-700 bp and 700-1300 bp inserted-size. The libraries were sequenced in a single lane of Illumina HiSeq 2000 platform. The sequence read data were deposited in the NCBI Sequence Read Archive (SRA) and is available under accession number SRR3741672. De novo assembly of the Illumina paired-end reads was performed using the software Hecate (unpublished, http://bgi-international.com/us/) using different k-mer sizes (41, 45, 49 and 63). The result of the 45-mer run provided the assembly with the best sequence coverage and N50 size, and therefore was determined to generate 4VS scaffolds.

### Detection of repeats and non-protein coding DNA

RepeatMasker software (version open-4.0.5, http://www.repeatmasker.org/) was used to detect repeat regions and masked repetitive DNA across the 4VS assembly sequence with WU-blast algorithm. Repetitive sequences were searched by aligning our sequence against the known repeats library Repbase Update [31] (http://www.girinst.org/repbase/) as well as TREP database (http://wheat.pw.usda.gov/ITMI/Repeats), using default settings.

To detect ribosomal DNA (rDNA) regions, a homology search against unmasked contigs using BLAT was performed with the options ‘-fine -q ¼ rna –out ¼ blast’ and thresholds of 95.0% identity and 100 bp coverage. As queries, four rDNA sequences, 5S (3IZ9), 5.8S (3IZ9), 18S (3IZ7), and 28S (3IZ9), the transfer RNA (tRNA) genes were predicted using the tRNAscan-SE version 1.3.1 program. The miRNA prediction was performed following the procedure in a previous report for wheat chromosome 6B [22].

### Scanning of coding sequences in the repeat-masked 4VS scaffolds

Ab initio gene prediction was performed by the AUGUSTUS program [32] using the repeat-masked sequences. The transcriptome data containing 204,258 unigenes that were compiled from leaves and endosperm of *H. villosa* (unpublished) were used to support the presence of the loci with these coding genes. We blasted the predicted gene sequence against the transcriptome data of *H. villosa* with e-value ≤ 10^−5^. Predicted genes with more than 90.0% identity and a minimum alignment of 200 bp on a “unigene” of transcriptome were defined as ‘evidenced genes’.

For GO analysis, we used Blast2GO [33] program to get GO annotation and WEGO [34] software for GO functional classification to understand the distribution of gene functions at the macro level.

### Identification a gypsy type retrotransposon and development of a probe specific for *H. villosa* chromosomes

By comparison of the assembled 4VS sequence and Chinese Spring reference release (IWGSC1 + popseq), a gypsy type retrotransposon RLG-Amy-contig1237 was identified (Additional file 1). RLG-Amy-contig1237 has 1362 copies in 4VS, while not found in the Chinese Spring. We speculate this is a repetitive sequence specifically present in *H. villosa*. The transposable element (TE) of RLG-Amy-contig1237 was selected to develop a cytogenetics marker for identification of *H. villosa* chromosomes. The procedure for the development of probe *pHv-Gypsy1* was as follows: According to the TE sequences, primer pair 4 Vrp2-F (gtccctggtgatgaatgtcc) and 4 Vrp2-R (gcctggagttttctgagctg) were designed and used to amplify genome DNA of *H.villosa*. The PCR procedure is: 3 min at 94 °C; 34 cycles of 30 s at 94 °C, 50 s at 55 °C, and 1 min and 10S at 72 °C; followed by 10 min at 72 °C. Amplification products were separated in 1.0% agarose gels. The expected amplicons were recovered from gels using DNA purification kit (Axygen, China), ligated into the plasmid vector pMD18-T (TaKaRa, Japan), and positive clones were sequenced for validation.

### Genomic in situ hybridization (GISH) and fluorescence in situ hybridization (FISH) analysis

Chromosome preparations of root tip cells at mitotic metaphase followed that of Chen et al. [35]. The techniques of GISH and FISH followed those of Zhang et al. [36]. Total genomic DNA of *H.villosa* was labeled with fluorescein-12-dUTP by Nick Translation method and used as a probe for GISH. The plasmid *pHv-Gypsy1* was labeled with digoxigenin-11-dUTP by Nick Translation method was and used as probes for FISH. Hybridization signals were observed using Olympus BX60 fluorescent microscope. Photographs were taken with SPOT CCD camera (Olympus DP72).

### Identification of syntenic regions in *Brachypodium*, rice and sorghum

To identify syntenic regions in the three model genomes, all the 1977 gene sequences predicted from *H. villosa* chromosome 4VS scaffolds were compared by blastn search against the coding sequence (CDS) database of *Brachypodium*, rice and sorghum (http://plants.ensembl.org/index.html). The following filtering criteria were applied: the first blast hits showing at least 70.0% identity and a minimum alignment of 200 bp were considered to be homologous [37].

### Virtual gene order map of *H. villosa* chromosome 4VS

The synteny between *Brachypodium*/rice/sorghum and *H. villosa* can be used to develop a linear gene order model of chromosome arm 4VS by Genome Zipper approach [19]. After testing, we choose *Brachypodium* as the reference zipper as it is considered the most closely related grass to wheat species. First we ordered 4VS genes based on their co-linearity to the *Brachypodium* reference genomes. The 4VS contained 785 genes in six regions of chromosomes 1 and 4 of *Brachypodium*. The orientations and the orders of these detected six regions were determined by referring to *H. villosa* 4VS “bin” map with a total of 26 markers within 13 different regions (physical bins) of 4VS [28]. The sequences for the 26 markers were blastn searched against the genes of *Brachypodium* along the chromosomes 1 and 4. All syntenic regions (having blastn hits) associated with markers were anchored to the *H. villosa* 4VS “bin” map and ordered following the concept of synteny and closest evolutionary distance.

## Results

### Shotgun sequencing and assembling of *H. villosa* chromosome 4VS

**Fig. 1:**
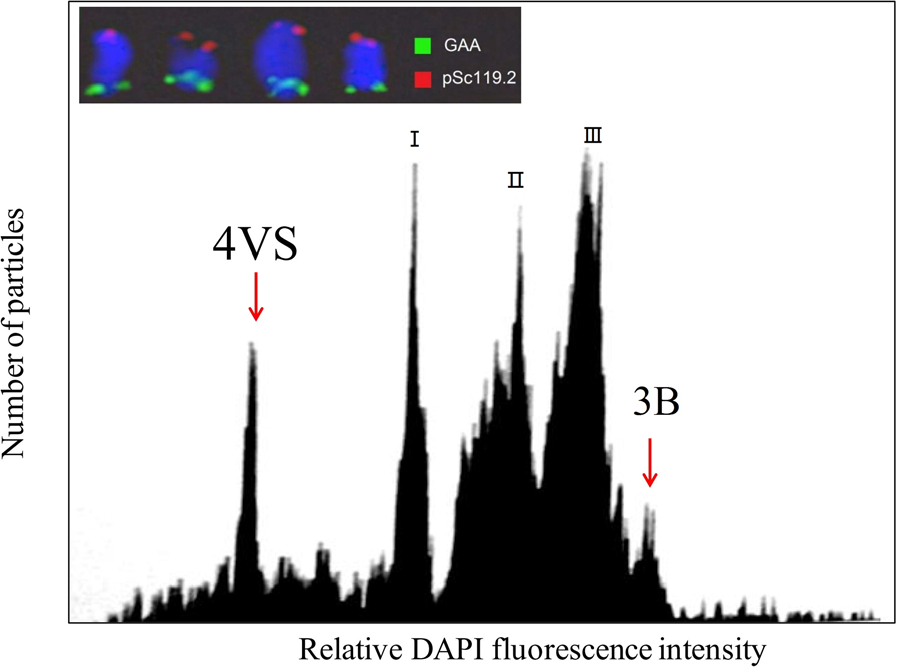
Histogram of the flow cytometric analysis of mitotic metaphase chromosomes of *T. aestivum*-*H. villosa* ditelosomic additional line Dt4VS. Peak corresponding to telosomes 4VS (red arrow) is well discriminated, which facilitated their flow sorting. Sorted chromosome arms were validated by fluorescence in situ hybridization (FISH) analysis using GAA (green) and pSc119.2 (red) as probes, which results in characteristic banding pattern (inset)

The DAPI-based flow karyotypes of wheat-*H. villosa* ditelosomic addition line Dt4VS showed 5 peaks (Fig. 1). By comparing with wheat variety Chinese Spring, the leftmost represented the peak of 4VS, which was well resolved from chromosome composite peaks I, II, III and peak of chromosome 3B of common wheat (Fig. 1). A total of 143,000 4VS arms were sorted in two batches. The flow sorted 4VS chromosomes were identified by FISH using probes GAA and *pSc119.2* (Fig. 1). The result showed that the fraction of 4VS ranged from 87.8% to 89.0% (data not shown). The contaminated fractions were a random mix of chromosomes and chromatid fragments. After DNA purification, 48.4 ng of chromosomal DNA was obtained from 143,000 flow-sorted 4VS arms with one arm is 0.338 pg (the 4VS chromosome is predicted to be 330.6 Mb in size), and was used for MDA as described by Šimková et al. [30]. The yield of amplified 4VS DNA was 23.0 μg.

After sequencing of 4VS DNA on Illumina platform, a high-quality of 33.5 Gb paired-end reads (each read being 90 bp, PE90) were generated from 2 libraries, with insert sizes ranging 400-700 bp and 700-1300 bp, respectively. De novo assembly was performed using the software Hecate (http://bgi-international.com/us/, unpublished) with different k-mer sizes (41, 45, 49 and 63). The result of the 45-mer run provided the assembly with the best sequence coverage and N50 size, and therefore was used to generate the 4VS scaffolds. The sequencing data and detailed assembly for 4VS are summarized in Table 1. A total length of 170.6 Mb assembled sequences was obtained, comprising 201,193 scaffolds. The maximum and minimum length of the scaffolds were 521,059 bp and 111 bp, respectively, with an N50 length (minimum length of scaffolds representing 50% of the assembly) of 59,654 bp and mean length of 848 bp. The length for each scaffold and the “N” content is summarized (Additional file 2: Table S1).

**Table 1:** The statistics for raw data and sequence assembly of *H. villosa* 4VS

4VS	Number
Total reads (PE90)	372,217,319
Total bases (Gbp)	33.5
Number of assembly scaffolds	201,193
Total assembly bases (bp)	170,640,133
Max. length of assembly scaffolds (bp)	521,059
Mini. length of assembly scaffolds (bp)	111
N50 (bp)	59,654
Mean length (bp)	848
GC-content (%)	47.5

### Repetitive DNA composition of 4VS and identification of a repetitive sequence specific for *H. villosa*

The overall repetitive DNA composition including transposable elements (TEs) and tandem repeats across the 4VS assembly was analyzed. When compared with two repeat databases combined, the Repbase Update library and the TREP library, a total of 64.6% of the 4VS assembly was corresponding to repeat elements. The retrotransposon LTR family composed about half of 4VS assembly (50.5%), followed by the DNA transposon (3.7%) and retrotransposon LINE (1.7%) (Table 2). Genomic content of TEs family in 4VS was compatible to that observed in wheat genome [21, 22, 38, 39]. Tandem Repeat Finder (TRF) was used to search for 51,171 tandem repeats which composed 9.5% of the 4VS assembly (Table 2).

**Table 2:** General feature of *H. villosa* 4VS assembly

Type	Sub-type	Number	Average length (bp)	Total length (bp)	% in 4VS
Protein coding gene					
Evidenced gene	–	1977	1301	2,571,967	1.51
Non-coding sequence					
miRNA	–	386	124.4	48,009	0.03
tRNA	–	121	74.3	8995	0.01
rRNA		0	0	0	0
	18S	0	0	0	0
	28S	0	0	0	0
	5.8S	0	0	0	0
	5S	0	0	0	0
snRNA		37	112.8	4175	0
	CD-box	23	108.9	2505	0
	HACA-box	12	117.8	1414	0
	splicing	2	128	256	0
Repetitive DNA					
DNA transposon	–	–	–	4,827,926	3.69
Retrotransposon					
	LTR	–	–	65,945,121	50.47
	LINE	–	–	2,171,029	1.66
	SINE	–	–	14,703	0.01
	Other	–	–	2711	0
	Unknown	–	–	135,142	0.10
Tandem repeat	–	51,171	214.0	12,349,526	9.45

*tRNA* transfer RNA; *rRNA* ribosomal RNA; *snRNA* small nucleolar RNA; *TEs* Transposable elements; *LTR* long terminal repeat; *LINEs* long interspersed nuclear elements; *SINEs* short interspersed nuclear elements

**Fig. 2:**
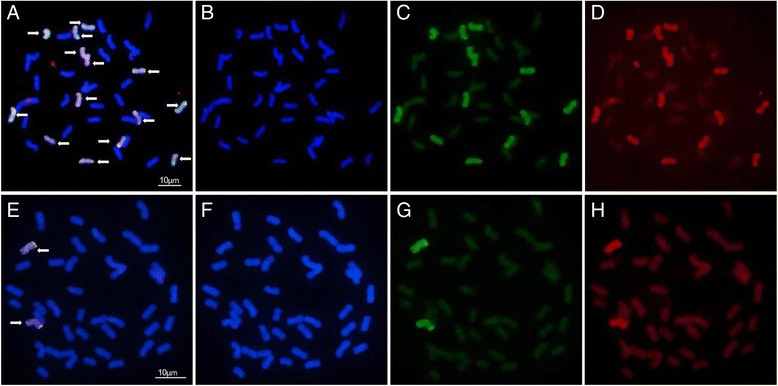
FISH using *pHv-Gypsy1* as probe, confirming its specificity to *H. villosa* chromosomes. The arrow indicated the chromosomes of *H. villosa*. **a**, **b**, **c** and **d** showed chromosomes of *T. durum-H.villosa* amphiploid (AABBVV) and E, F, G and H showed chromosomes of common wheat-*H. villosa* disomic substitution line DS3V. **a** Merged image from **b**, **c** and **d**; (**b**) 4′,6-diamidino-2-phenylindole (DAPI) stained chromosomes; (**c**) GISH analysis using *H. villosa* genome DNA as probe (Green), the 14 *H. villosa* chromosomes were shown green; (**d**) FISH analysis using *pHv-Gypsy1* as probe (Red), the 14 *H. villosa* chromosomes were shown red. **e** Merged image from **f**, **g** and **h**; (**f**) DAPI stained chromosomes; (**g**) GISH analysis using *H. villosa* genome DNA as probe (Green), the pair of chromosome 3 V were shown green; (**h**) FISH analysis using *pHv-Gypsy1* as probe (Red), the pair of chromosome 3 V were shown red

The assembled 4VS sequence and Chinese Spring reference release (IWGSC1 + popseq) were compared for their difference in copy numbers in the corresponding genomes. A gypsy type retrotransposon RLG-Amy-contig1237 has 1362 copies in 4VS, while none was found in Chinese Spring (Additional file 1). We speculate this is a repetitive sequence specifically present in *H. villosa*. To validate the 4VS assembly,RLG-Amy-contig123 was designed as a plasmid FISH probe *pHv-Gypsy1*. GISH using *H. villosa* genome DNA followed by FISH using *pHv-Gypsy1* as probe was performed in the *T. durum-H.villosa* amphiploid (AABBVV). The FISH signals by *pHv-Gypsy1* were only observed on all the 14 chromosomes *of H. villosa* VV genome, while was not detected on any of the 28 chromosomes of *T. durum* AA or BB genomes (Fig. 2, a-d). Further FISH using *pHv-Gypsy1* in wheat-*H. villosa* substitution line DS3V showed that no FISH signal was observed on any wheat chromosomes (Fig. 2, e-h). This confirmed our prediction that the *pHv-Gypsy1* was *H. villosa*-specific. *pHv-Gypsy1* can be used as a cytogenetic marker to differentiate chromosomes of *H. villosa* from those of common wheat background.

### Non-protein coding DNA sequences

A total of 386 different putative miRNAs and 121 tRNA genes were identified in the 4VS scaffolds (Table 2). No rRNA genes were detected, which is consistent with the previous result that 45S rDNA and 5S rDNA loci was not present on 4VS. Other non-protein coding DNA, such as snRNA was also identified, including three types, CD-box, HACA-box and splicing (Table 2).

### Protein coding genes

The repeat-masked sequences of 4VS were used for Ab initio gene prediction by AUGUSTUS program which identified a total of 12,762 loci of predicted coding sequences. The unigenes from the high-throughput RNA-seq data from leaves and endosperm of *H. villosa* were used as expression evidences for supporting the presence of the predicted coding loci. The detailed criterions were described in the Material and Methods. Using both Ab initio and evidence-based gene predictions, we finally identified a total of 1977 high-confidence protein-coding loci on 1069 scaffolds of chromosome 4VS (Table 2; Additional file 2: Table S2). The gene length distribution is shown by Additional file 3: Figure S1. These genes composed of a total length of 2,577,795 bp, which accounts for 1.5% of 4VS assembly genome. This estimate is compatible with the gene content annotated in the reported wheat genome or chromosomes [4]. A total of 985 genes were functionally assigned to one or more Gene Ontology (GO) terms (Additional file 3: Figure S2). To summarize, 425, 787 and 718 genes were annotated with cellular component, molecular function and biological process, respectively. There are some functional categories enriched with 4VS annotated genes, such as cell part localization (144, 25.7%), binding (380, 67.9%) and metabolic related (266, 47.5%), et al.

### Comparative analysis of genome sequence of 4VS

**Fig. 3:**
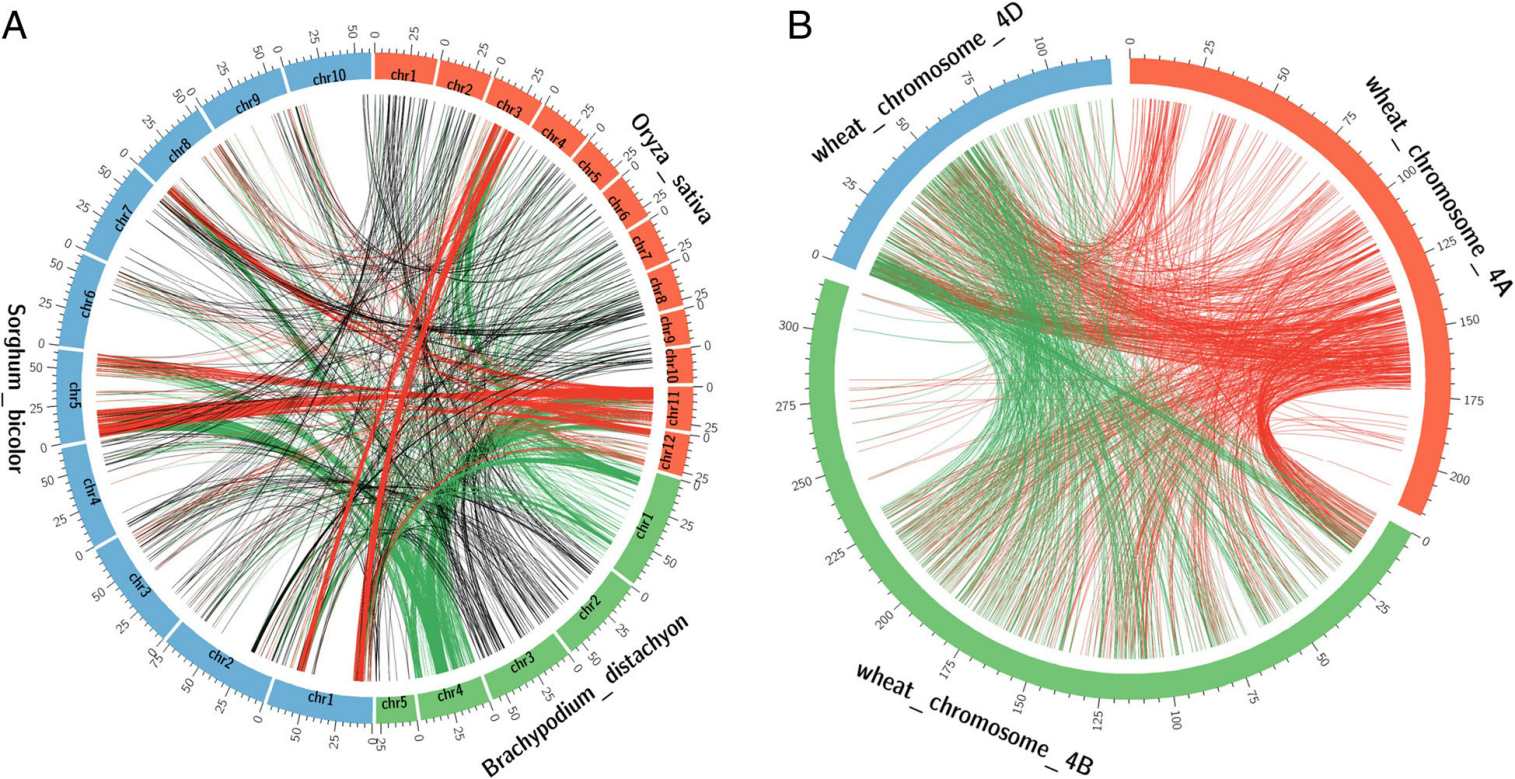
The circos map of the syntenic regions for *H. villosa* 4VS annotated genes. **a** Syntenic regions among *B. distachyon*, rice and sorghum were represented by homologous gene pairs between two of the species. Only the gene pairs having homologs in 4VS were shown in the figure. The homologous loci on chromosomes 1 and 4 of *Brachypodium* were shown as green links, on chromosomes 3, 11 and 12 of rice, and chromosomes 1, 5 and 8 of sorghum were shown as red and green links, respectively. The homologous loci on other chromosomes were shown as black links; (**b**) Syntenic analysis among 4VS, and wheat chromosomes 4A, 4B and 4D. The homologous loci on wheat chromosome 4A were shown as red links, on 4B and 4D shown as red and green links, respectively. The tick labels on the cycle outline mean million base pairs; chr: chromosome

With the availability of genome sequences of *Brachypodium*, rice and sorghum (http://plants.ensembl.org/), all 1977 gene sequences predicted from *H. villosa* 4VS scaffolds with transcriptional evidence (evidenced genes) were used to identify syntenic regions in genomes of other grass species. After filtering (Materials and Methods), a total of 942 out of these 1977 (47.6%) 4VS evidenced genes had blastn hits to the genes in at least one of three species *Brachypodium*, rice and sorghum, with gene number of 922, 878 and 890, respectively (Additional file 3: Figure S3A). In other word, these 942 annotated 4VS genes were also evidenced by at least one of the reference organisms. Moreover, 840 out of 942 (89.2%) identified homologous genes were shared among all the three reference genomes. This suggested their close phylogenetic relationship and the existence of collinear regions between *H. villosa* 4VS and other three species. The 942 4VS homologous genes were plotted according to their positions on the chromosomes of their respective species, and clear syntenic regions among these species were observed (Fig. 3). The 4VS syntenic regions in rice, *Brachypodium* and sorghum were distributed on Rice chromosomes 3, 11 and 12 (Fig. 3a, red links), *Brachypodium* chromosomes 1 and 4 (green links), and sorghum chromosomes 1, 5 and 8 (red links). These results were in agreement with other comparative studies using wheat chromosome 4AS and 4DS [21, 24] which are homologous chromosomes of 4VS. We also observed syntenic regions on other chromosomes of the three species, but with low gene density (black links in Fig. 3). These regions probably result from either genes that moved from their original positions or false positive matches due to the alignment between paralogous genes.

We used a set of “toplevel” wheat sequences consisting of molecule-level assemblies (http://plants.ensembl.org/) that were released by IWGSC [8] to identify 4VS syntenic regions on wheat 4A, 4B and 4D. A total of 893 out of 1977 (45.2%) 4VS evidenced genes have blastn hits, with the number of homologous genes in wheat 4A, 4B and 4D was 574, 773 and 525, respectively (Additional file 3: Figure S3B). One of genes coding for A DELLA protein RHT1 (*wheatA12577*) was identified on 4VS. This is a homologues allele of wheat *Rht-1* genes on wheat 4A, 4B and 4D [40]. The syntenic genes of wheat 4A, 4B and 4D were plotted according to the position of their respective chromosomes, as to highlight the syntenic regions. Similarly, the syntenic regions with high genes density were observed on 4AL, 4BS and 4DS (Fig. 3, b). A high density of 4VS genes was homologous to those on 4AL, confirming the presence of evolutionary chromosome rearrangement in wheat [41].

### A virtual gene map of 4VS

**Fig. 4:**
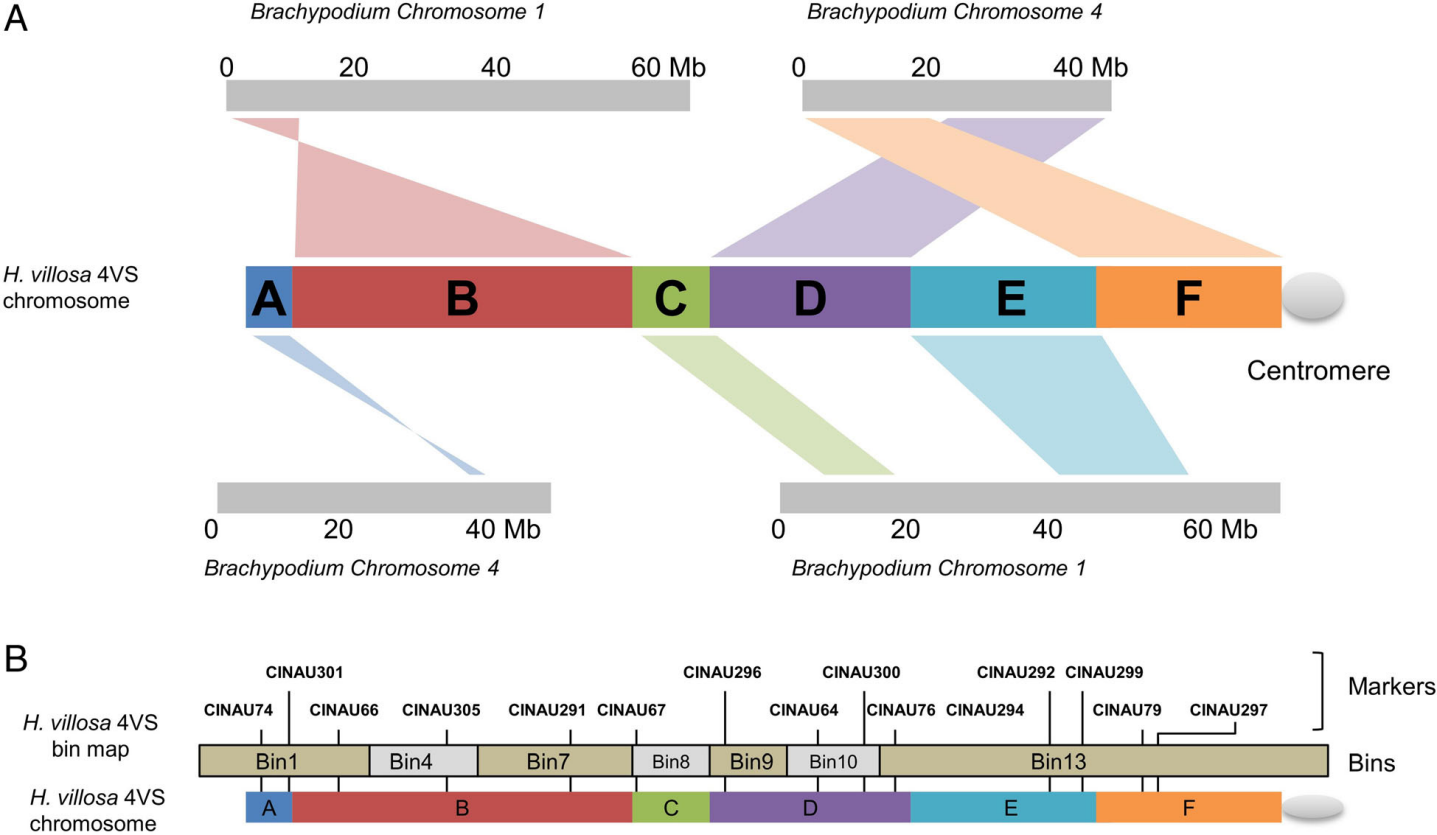
Comparison of the *H. villosa* 4VS synteny regions in *Brachypodium* genome. **a** The syntenic regions of 4VS (from A to F) corresponded to six genetic regions of chromosomes 1 and 4 of *Brachypodium*. **b** The orders and orientations of the syntenic regions of 4VS were determined by referring to 4VS physical bin map constructed by Zhao et al. [28]. Totally the physical map has 13 Bins. However, only 15 EST markers located in Bins 1, 4, 7, 8, 9, 10, 13 could find the homologous genes in *Brachypodium*. ESTs markers located in Bins 2, 3, 5, 6 11 and 12 failed to find homologous genes in *Brachypodium*. Mb: million base pairs

The syntenic regions in *Brachypodium*, rice or sorghum were used to define virtual gene order of 4VS and construct its physical map, by taking the advantage of the high colinearity among grass species using genome zipper approach [42]. The *Brachypodium* gene order was selected as a reference because *Brachypodium* is considered the most closely related species to wheat. As described above, the 4VS synteny was distributed on six different genetic regions (Fig. 4, from A to F) of *Brachypodium* chromosomes 1 and 4 (Fig. 3a, green links). Their orders and orientations were determined by referring to 4VS physical bin map constructed in our lab using a number of wheat-*H.villosa* translocation lines involving 4VS [28]. The 4VS physical bin map was constructed based on the presence/absence of amplicons of 4VS specific markers, which were designed according to the expressed sequence tags (EST) with known chromosome location on wheat homoeologous group 4 chromosomes. The sequences (all ESTs) of 15 4VS specific markers can be aligned to corresponding *Brachypodium* genes. Therefore, the physical bin map along with these markers was used as a backbone to arrange the syntenic regions. Using this genome zipper approach [42], a final 4VS virtual gene map was obtained. Out of the 1, 977 evidenced genes, 785 genes corresponding to the *Brachypodium* chromosomes 1 and 4 were mapped to physical region of 4VS (Fig. 4; Additional file 2: Table S3).

## Discussions

Draft sequence of the short arm of *H. villosa* chromosome 4V was obtained by flow-sorted and next generation sequencing. The molecular organization of this arm was revealed by identifying repetitive sequences, protein-coding genes, non-protein-coding tRNA and miRNA genes, rDNA regions. We have also determined the syntenic relationships with genomes of other grass species. A final assembly of 170.6 Mb consisting of 201,193 scaffolds was obtained with an N50 length of 59,654 bp. Among the scaffolds, 1661 were larger than 10,000 bp representing 66.9% of the assembly, which implied a high quality of assembly of 4VS. The content of TE families was 55.5%, which is lower than that in *Ae. tauschii* genome (65.9%) [5] or that in chromosome 3B (82.0%) [9]. This may be explained by collapsing repetitive regions when assembling short reads obtained by NGS technologies [43].

The annotation of protein-coding genes with evidence-based quality indexing has been proved the reliable method for the prediction of genes of high-confidence [21] and has been implemented in the automatic annotation pipeline TriAnnot [44]. Using the similar methods, in the present research, Ab initio gene prediction by AUGUSTUS program estimated a preliminary of 12,762 coding sequences (data not show). To aid in gene identification, we used transcriptome data obtained from leaf and endosperm to perform a rather stringent comparison between the predicted genes and “unigenes” in the transcriptome. Combining these two methods, we identified 1977 evidenced genes. The average density of genes content was 11.6 genes per Mb or one gene per 86.3Kb. The gene number and the density are similar to those of wheat chromosome arms [8]. After aligning to genes in three grass genomes and wheat chromosomes 4A, 4B, 4D [8], a total of 1, 034 out of 1977 evidenced genes had their homologous genes in at least one of these species (data not shown). Because of the stringent criterions used for identification of homologous genes, the remaining genes with no homologies (943 genes) may be due to the low similarities of these genes to those of the species. Moreover, the homologous genes only corresponded to 34.1%, 57.3% and 68.7% genes of 4AL, 4BS and 4DS, respectively (Additional file 3: Figure S3B), suggesting these evidenced genes may not well represent all 4VS genes. This makes sense because on one hand, the genes of low similarity (943 genes) were excluded from consideration of comparison with wheat genes; on the other hand, the reference “unigenes” assembled only from two tissues of *H. villosa* may cause biased gene expression profiling. More RNA-seq data from more tissues will be needed to obtain a better comprehensive gene content of 4VS.

The establishment of gene order along a chromosome is of importance for mapping, positional gene cloning and physical map anchoring [21]. In general, despite divergence, the synteny has been reported to be retained among Poaceae species [45, 46]. Therefore, by referring to the collinear order of the genes from the model species as reference, this synteny allowed the placement of the identified genes of 4VS in a probable order along chromosome as suggested by “GenomeZipper” approach [19]. As 4VS evidenced genes matched the most numbers of genes in *Brachypodium* (Additional file 3: Figure S3), we generate a virtual gene order for 4VS using *Brachypodium*as “genome zipper” as reference (Fig. 4). Chromosome 4A undergone structural re-arrangement including pericentric inversion during wheat evolution [47], no chromosomal rearrangements were reported on 4D. As a member of the same homoeologous group, *H. villosa* chromosome 4VS should share similar structural features, especially with 4D in genes contents and their linear order. By taking the advantage of the release of *Ae. tauschii* genetic map, we generated a “genome zipper” containing 1137 4VS genes by referring to 4D scaffolds which was anchored to chromosome 4D of *Ae. tauschii* (Additional file 2: Table S4). The two 4VS “genome zipper” versions were compared and they were correlated (Additional file 3: Figure S4). This indicated the accuracy of “genome zipper” approach for determination of 4VS virtual genes order. However, some inconsistencies were detected, mainly in the bin from 50 to 60 of *Ae. tauschii* 4D genetic map, where the centromeric region located. The inconsistencies was also detected for 4D [21] indicating this special region experienced chromosomal rearrangements during evolution.

The sequence information in this study can be directly used to identify candidate genes underlying important agronomic traits on 4VS and develop DNA markers linked to these genes. We mapped wheat spindle streak mosaic virus (WSSMV) or WYMV resistance gene, *Wss1* to 4VS [26]. By the development of more translocations involving 4VS Zhao et al. physically mapped the *Wss1* to a narrowed specific chromosome region [28]. However, due to the lower density of markers used in determination of translocated alien fragments, they only generated a physical maps with limited resolution, which consisting of 13 bins. Facilitated by the availability of 4VS scaffolds, a total of 235 PCR-based STS markers were developed [29] which will dramatically increase the physical map density. Once the resistant gene was fine-mapped, the synteny-based 4VS genome-zipper will be especially helpful for resistance candidate genes prediction. A seed storage protein gene (*wheatA13227*) coding for alcohol dehydrogenase-1 (ADH-1) was identified. This gene showed a homologues allele variance with wheat genes at protein level [48] which may affect grain protein quality. A DELLA protein RHT1 (*wheatA12577*), which is a homologues allele of wheat *Rht-1* gene, was identified [40]. Therefore, using 4VS small fragment translocation lines we can study whether this gene can affect wheat plant height so as to evaluate its potential use in breeding. We also found lipoxygenase 1 gene (*Lpx-1*) in 4VS scaffold (Hecate_CTG:136,974,658,917,422,189) but not in 4VS evidenced genes. This could be explained by the previous report that the gene in *H. villosa* does not show clear Lpx-1 activity [48]. Therefore, the gene was not included because annotation of 4VS genes was expression-supported. The aminopeptidase (AMP-2) gene was reported on 4VS chromosome homoeoloci [49]. In the present study, this gene could be identified in 4VS scaffold (Hecate_CTG:136,974,920,910,404,953) rather than in 4VS genes due probably to low level of AMP-2 activity detected in young leaves and endosperm. We also found homologous allele of protein disulfide isomerase (PDI) [50] in 4VS genes (*wheatA05752*), which was evolved in seed germination [51]. These indicate that the assembled 4VS genome sequences will accelerate high-throughput gene mining. Our work also provides an example for genome sequencing of the remained *H. villosa* chromosomes.

## Conclusion

Here we provide valuable genetic information obtained after shotgun sequencing flow-sorted short arm of *H. villosa* chromosome 4V. In silico prediction along with evidenced transcriptome data identified 1977 gene loci of high-confidence. Comparative genomic analysis showed higher level of synteny with wheat group 4 chromosomes, *Brachypodium* chromosomes 1 and 4, rice chromosomes 3 and 11, and sorghum chromosomes 1, 5 and 8. The genome-zipper based gene order data will serve as a valuable resource for DNA marker development, positional gene cloning and physical map anchoring, especially for the wild species *H. villosa* with scarce data on genome organization. Moreover, a comprehensive understanding of chromosome arm 4VS could be a model for future sequencing of its entire genome using the similar strategy.

## Additional files

Additional file 1: Sequence of LTR Gypsy-type TE RLG-Amy-contig1237. (PDF 134 kb)

Additional file 2: Table S1. The scaffold length distribution; **Table S2.** The numbers of genes in a scaffold; **Table S3.** Genome Zipper-based gene orders of *H. villosa* chromosome 4VS in *Brachypodium*; **Table S4.** Genome Zipper-based gene orders of *H. villosa* chromosome 4VS in *Ae. tauschii* chromosome 4D. (ZIP 4749 kb)

Additional file 3: Figure S1. Sequence length distribution of 1977 genes of *H. villosa* chromosome 4VS. **Figure S2.** Percentage distribution of the GO entries for *H. villosa* 4VS genes. The most represented entries within the three ontologies (Molecular function, Biological process and Cellular component) are indicated. **Figure S3.** Synteny between chromosomes of *H. villosa* and other species. (A) Conservation of synteny between *H. villosa* chromosome 4VS and Brachypodium (*B. distachyon*), rice (*Oryza sativa*) and sorghum (*Sorghum bicolor*). (B) Conservation of synteny between *H. villosa* chromosome 4VS and wheat chromosome 4A, 4B and 4D. The Venn diagrams display the numbers of genes shared between 4VS and one reference genome (outer cycle), and the number of shared conserved genes among the three grass genomes (inner cycle). **Figure S4.** Comparison of the 4VS genome zipper based on Brachypodium chromosomes 1 and 4 with *Ae. tauschii* chromosome 4D. Y-axis: the virtual 4VS gene order is marked from 1 to 785; X-axis: the corresponding scaffold in bins. (PPTX 966 kb)

## Abbreviations

AMP-2: aminopeptidase
DAPI: 4′, 6-diamidino-2-phenylindole
EST: expressed sequence tags
FISH: fluorescence in situ hybridization
GISH: genomic in situ hybridization
GO: Gene Ontology
LINEs: long interspersed nuclear elements
Lpx-1: lipoxygenase 1 gene
LTR: long terminal repeat
MDA: multiple displacement amplification
miRNA: microRNA
PDI: disulfide isomerase
PE: pair-end
rDNA: ribosomal DNA
SINEs: short interspersed nuclear elements
snRNA: small nucleolar RNA
TE: transposable element
TRF: Tandem Repeat Finder
tRNA: transfer RNA
WSSMV: wheat spindle streak mosaic virus
WYM: wheat yellow mosaic
