# Supplementary material for: Sequencing flow-sorted short arm of Haynaldia villosa chromosome 4V provides insights into its molecular structure and virtual gene order
Source: BMC Genomics. 2017 Oct 16;18:791. doi: 10.1186/s12864-017-4211-7 (PMC5644170; doi:10.1186/s12864-017-4211-7)
Supplement: Supplementary file 1 — Sequence of LTR Gypsy-type TE RLG-Amy-contig1237. (PDF 134 kb) [file 12864_2017_4211_MOESM1_ESM.pdf]

> RLG-Amy-contig12371362 LTR.Gypsy similar

gtcgaccgccaaggaccatccgagcaccaccgtagatctgcagcttcgccggaccgccgtgccgactcgactccgacgagaacttcaac
tccggtatgtcttctgtcgccgcgagttttcatcgttgatacctttcctcatatcttgtctgctgcacagcttctccatgaataccatgagttcatacc
tttatactgtgttcatagcaaagtttaaatttcttttctgctctgccgtagatctttagcttccagtaaacattgtgaaacctgtttcactgctcccttta
gacttagcaaaaacttcagtagagatgtagactgagaaggccggtttatgtgccgccacctatctcattagccctcagtttaaaccgtctgtta
gcgaaacgactttaaaccggaaatcattcatgtgtagcataataaaccggtagccgaaacttctagctgttaacccggacatatatgcctttcc
ctttaatccgtcagccgcaaattctagctgataacccggaaatatatgcctttccctttaatccgtcagccgcaaattctagctgataatccggaa
atttgtacccttcctttaaaccgtcagccgaaatttctagctgcgttaacattgtcttgcaataatattgttatattccttccgatttaacatactgtaa
gtgtacttaaaccggaaatttccaccctctgcagtcaatggcaacttacgcaaatgactgggctccatcccgggttaccgagggagctttgat
cgacttagtgaaccagggctggttacccagtaaggatgccataggctggcgtgtccctggtgatgaatgtccaccaactccagctgaagga
gaagtcattgtttttgttgaccacttgtaccggggtttctctccccccggttcaaagttctttcgggatgctctgaattaccttcagctgcatccgc
aagacattggacctaattctgtttccaacctctgcaacttccaagtatttgctgaagcttacctattagaggagccatccattgagatgttcaggg
atttcttctatttaaaccggcgcaccgagtttggaaaaggcccctgtactatgctaggaggaatggcgattcaaaaacgccggtctgtacaatt
tccgcacgccactctgcacagtcatccgaaagaatggaatagcacctggttttattgcagagacacgtctccaaccgatgagaaccggttgc
cgggttaccgtgctgacagacttaacccggagcatccaatgccacaacagctatcagcacctgaaagggctcaattcattcctcagctcaga
aaactccaggcatatatcaacaatggtttaa
