# Supplementary material for: Sequencing flow-sorted short arm of Haynaldia villosa chromosome 4V provides insights into its molecular structure and virtual gene order
Source: BMC Genomics. 2017 Oct 16;18:791. doi: 10.1186/s12864-017-4211-7 (PMC5644170; doi:10.1186/s12864-017-4211-7)
Supplement: Supplementary file 3 — Sequence length distribution of 1977 genes of H. villosa chromosome 4VS. Figure S2. Percentage distribution of the GO entries for H. villosa 4VS genes. The most represented entries within the three ontologies (Molecular function, Biological process and Cellular component) are indicated. Figure S3. Synteny between chromosomes of H. villosa and other species. (A) Conservation of synteny between H. villosa chromosome 4VS and Brachypodium (B. distachyon), rice (Oryza sativa) and sorghum (Sorghum bicolor). (B) Conservation of synteny between H. villosa chromosome 4VS and wheat chromosome 4A, 4B and 4D. The Venn diagrams display the numbers of genes shared between 4VS and one reference genome (outer cycle), and the number of shared conserved genes among the three grass genomes (inner cycle). Figure S4. Comparison of the 4VS genome zipper based on Brachypodium chromosomes 1 and 4 with Ae. tauschii chromosome 4D. Y-axis: the virtual 4VS gene order is marked from 1 to 785; X-axis: the corresponding scaffold in bins. (PPTX 966 kb) [file 12864_2017_4211_MOESM3_ESM.pptx]

## Slide 1
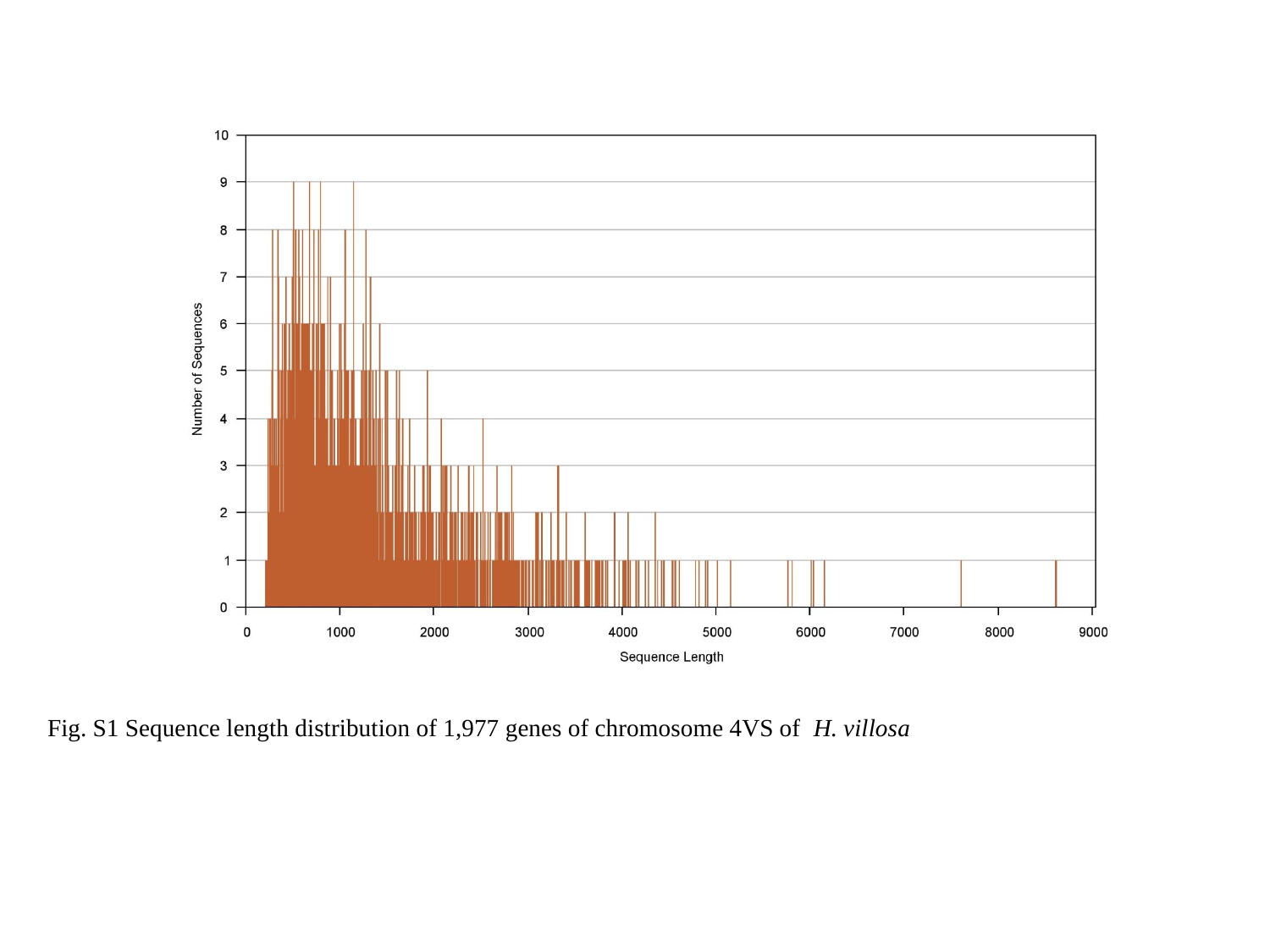

Fig. S1 Sequence length distribution of 1,977 genes of chromosome 4VS of H. villosa

## Slide 2
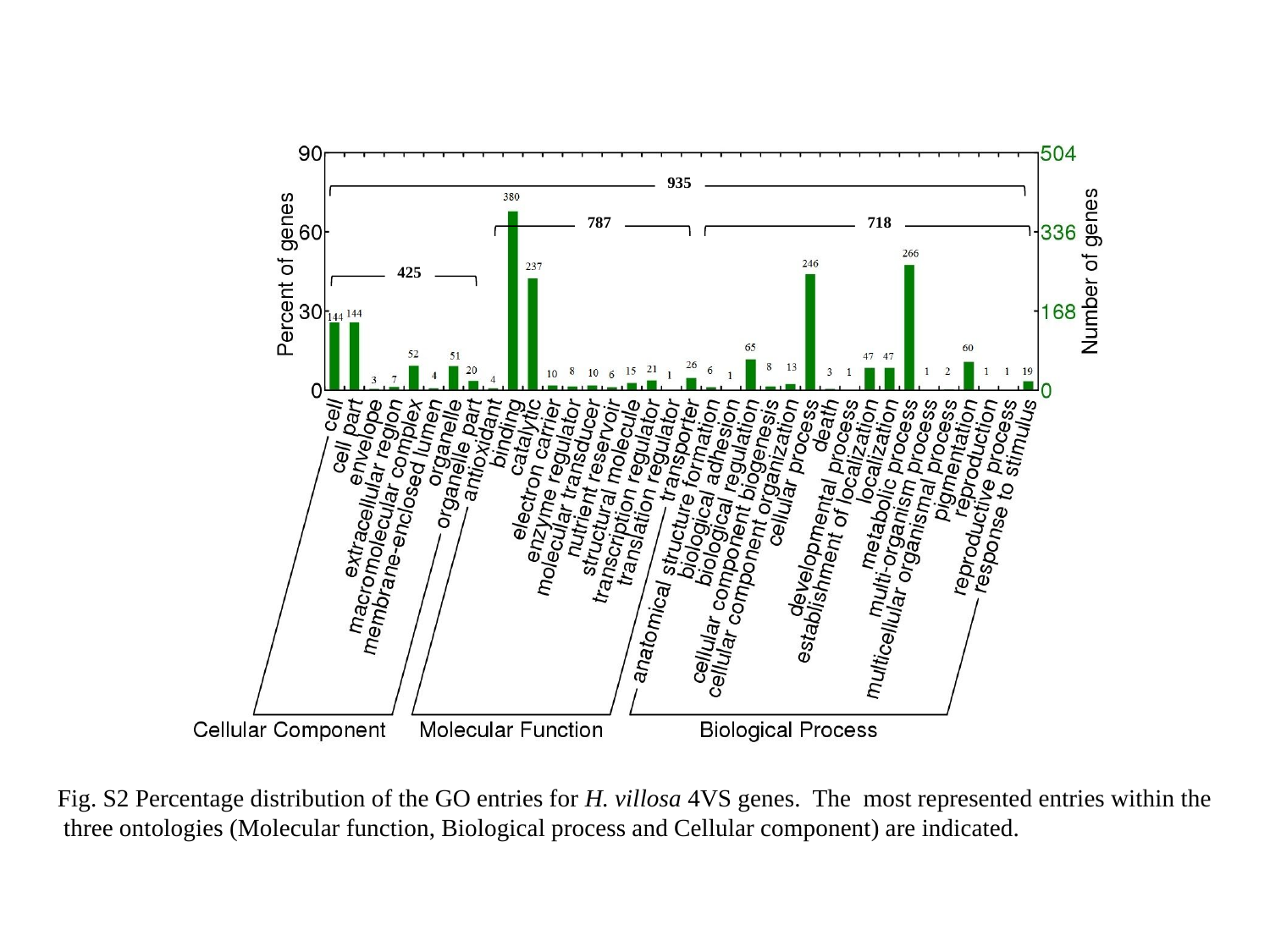

935
787
718
425
Fig. S2 Percentage distribution of the GO entries for H. villosa 4VS genes. The most represented entries within the three ontologies (Molecular function, Biological process and Cellular component) are indicated.

## Slide 3
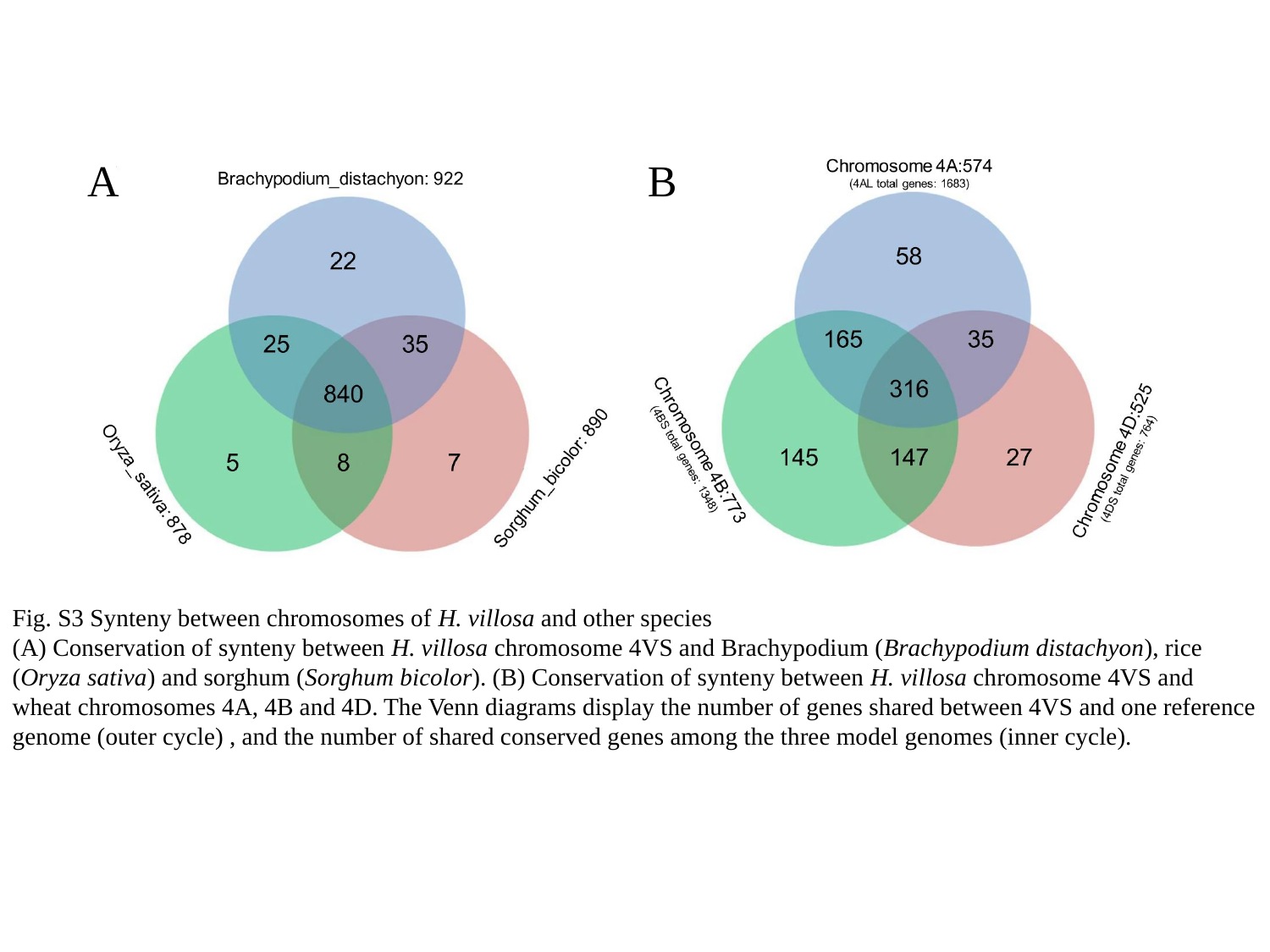

A
B
Fig. S3 Synteny between chromosomes of H. villosa and other species
(A) Conservation of synteny between H. villosa chromosome 4VS and Brachypodium (Brachypodium distachyon), rice (Oryza sativa) and sorghum (Sorghum bicolor). (B) Conservation of synteny between H. villosa chromosome 4VS and wheat chromosomes 4A, 4B and 4D. The Venn diagrams display the number of genes shared between 4VS and one reference genome (outer cycle) , and the number of shared conserved genes among the three model genomes (inner cycle).

## Slide 4
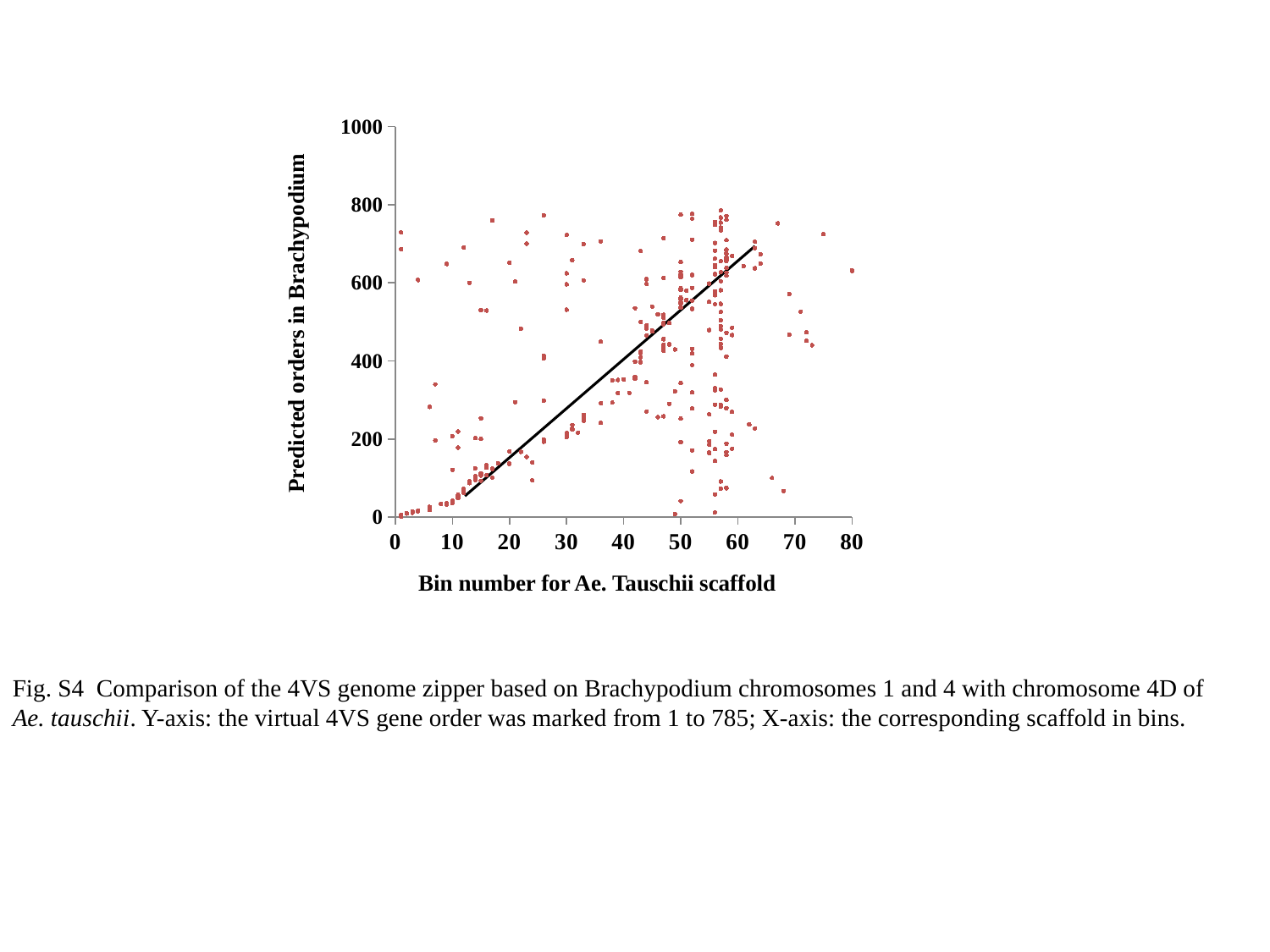

### Chart
| Category | |
|---|---|Predicted orders in Brachypodium
Bin number for Ae. Tauschii scaffold
Fig. S4 Comparison of the 4VS genome zipper based on Brachypodium chromosomes 1 and 4 with chromosome 4D of Ae. tauschii. Y-axis: the virtual 4VS gene order was marked from 1 to 785; X-axis: the corresponding scaffold in bins.
